# Supplementary material for: A Likelihood Approach for Real-Time Calibration of Stochastic Compartmental Epidemic Models
Source: PLoS Comput Biol. 2017 Jan 17;13(1):e1005257. doi: 10.1371/journal.pcbi.1005257 (PMC5240920; doi:10.1371/journal.pcbi.1005257)
Supplement: S1 File — (TAR.GZ) [file pcbi.1005257.s014.tar.gz › HSPH_Online-SI-Revision/Additions/PF09/n100-extreme-SEITR/n100-extreme-SEITR_PF2_table0.pdf]

1	newly 3 weeks spec	6372.	6016.52	{5912., 6137.}	5.57878	0.024977	newly 3 weeks cumul	27578.	17908.4	{17735., 18038.}	35.0629	0.187519	newly 5 weeks spec	2747.	5222.56	{5119., 5330.}	90.1187	0.278971	newly 5 weeks cumul	34544.	28829.4	{28525., 29082.}	16.543	0.0785468	newly 51 weeks cumul	38811.	57098.1	{56736., 57420.}	47.1182	0.167662
2	newly 3 weeks spec	7321.	10385.	{10232., 10509.}	41.8526	0.151811	newly 3 weeks cumul	36564.	32973.2	{32794., 33142.}	9.82059	0.0448967	newly 5 weeks spec	2664.	7214.79	{7083., 7318.}	170.825	0.43266	newly 5 weeks cumul	43633.	49035.5	{48858., 49252.}	12.3816	0.0506929	newly 58 weeks cumul	47260.	73062.3	{72838., 73245.}	54.5965	0.189199
3	newly 3 weeks spec	8747.	10513.4	{10367., 10649.}	20.194	0.0798597	newly 3 weeks cumul	40258.	32331.9	{32152., 32518.}	19.6882	0.0952246	newly 5 weeks spec	3413.	7568.31	{7471., 7656.}	121.749	0.345843	newly 5 weeks cumul	49075.	49055.4	{48795., 49308.}	0.309608	0.00134535	newly 60 weeks cumul	54208.	74854.9	{74609., 75022.}	38.0882	0.140155
4	newly 3 weeks spec	8496.	9532.74	{9359., 9680.}	12.2027	0.0499664	newly 3 weeks cumul	36998.	32013.6	{31634., 32367.}	13.4721	0.0628604	newly 5 weeks spec	3304.	6301.49	{6186., 6396.}	90.7231	0.280367	newly 5 weeks cumul	45863.	46192.9	{45697., 46666.}	0.879707	0.00379811	newly 58 weeks cumul	50745.	66621.	{66027., 67111.}	31.2859	0.118209
5	newly 3 weeks spec	5120.	10740.9	{10615., 10872.}	109.784	0.321751	newly 3 weeks cumul	31803.	32727.9	{32530., 32908.}	2.90809	0.0124449	newly 5 weeks spec	1318.	7789.24	{7674., 7896.}	490.989	0.771553	newly 5 weeks cumul	35687.	49916.2	{49699., 50123.}	39.8721	0.145728	newly 60 weeks cumul	37045.	76486.3	{76283., 76665.}	106.469	0.314853
6	newly 3 weeks spec	4778.	10132.3	{9975., 10273.}	112.062	0.326439	newly 3 weeks cumul	31868.	32920.6	{32647., 33168.}	3.30287	0.0141046	newly 5 weeks spec	1057.	6900.39	{6793., 6996.}	552.828	0.814773	newly 5 weeks cumul	35197.	48361.9	{47992., 48659.}	37.4036	0.137993	newly 59 weeks cumul	35989.	70980.9	{70689., 71298.}	97.2293	0.294969
7	newly 3 weeks spec	7357.	6359.91	{6237., 6458.}	13.5529	0.0632928	newly 3 weeks cumul	28195.	19174.7	{18999., 19330.}	31.9926	0.167455	newly 5 weeks spec	3977.	5438.76	{5315., 5551.}	36.7553	0.135889	newly 5 weeks cumul	37670.	30598.7	{30353., 30828.}	18.7717	0.0903007	newly 51 weeks cumul	47209.	59056.	{58694., 59392.}	25.0948	0.0972343
8	newly 3 weeks spec	7216.	9903.87	{9766., 10026.}	37.2488	0.137488	newly 3 weeks cumul	32722.	32250.1	{32085., 32427.}	1.4423	0.00631301	newly 5 weeks spec	3068.	6732.42	{6600., 6831.}	119.44	0.341281	newly 5 weeks cumul	40537.	47324.2	{47176., 47525.}	16.7432	0.0672299	newly 58 weeks cumul	46063.	69562.8	{69395., 69704.}	51.0167	0.179024
9	newly 3 weeks spec	5164.	11210.7	{11055., 11348.}	117.093	0.336619	newly 3 weeks cumul	32212.	31432.4	{31165., 31695.}	2.42009	0.010648	newly 5 weeks spec	1330.	8869.43	{8750., 9003.}	566.874	0.824019	newly 5 weeks cumul	36196.	50637.7	{50323., 50958.}	39.8985	0.145808	newly 60 weeks cumul	37584.	82420.9	{82171., 82656.}	119.298	0.341033
10	newly 3 weeks spec	8694.	8849.96	{8698., 8984.}	1.88268	0.00807724	newly 3 weeks cumul	33638.	28455.6	{28266., 28654.}	15.4065	0.0726693	newly 5 weeks spec	4707.	6300.5	{6173., 6389.}	33.8538	0.126598	newly 5 weeks cumul	44803.	42371.9	{42155., 42591.}	5.42622	0.0242322	newly 56 weeks cumul	56292.	64943.7	{64729., 65125.}	15.3693	0.0620892
11	newly 3 weeks spec	8565.	7181.45	{7076., 7295.}	16.1535	0.076548	newly 3 weeks cumul	34772.	19212.7	{18991., 19419.}	44.7466	0.257656	newly 5 weeks spec	4255.	7214.28	{7095., 7328.}	69.5483	0.229261	newly 5 weeks cumul	45069.	33816.2	{33476., 34065.}	24.968	0.124763	newly 55 weeks cumul	54734.	74619.2	{74388., 74879.}	36.3307	0.134592
12	newly 3 weeks spec	7136.	8905.91	{8793., 8995.}	24.8026	0.096204	newly 3 weeks cumul	31568.	29070.8	{28915., 29236.}	7.9107	0.035795	newly 5 weeks spec	3139.	6207.64	{6094., 6310.}	97.7585	0.296099	newly 5 weeks cumul	39318.	42847.	{42674., 43000.}	8.97548	0.0373267	newly 56 weeks cumul	44760.	64235.4	{64085., 64386.}	43.5107	0.156884
13	newly 3 weeks spec	7637.	9482.44	{9329., 9607.}	24.1645	0.0939657	newly 3 weeks cumul	33066.	28959.3	{28761., 29132.}	12.4199	0.0575989	newly 5 weeks spec	3594.	7131.74	{7015., 7242.}	98.4346	0.297581	newly 5 weeks cumul	41882.	44529.1	{44325., 44722.}	6.3203	0.0266136	newly 57 weeks cumul	49021.	70889.3	{70715., 71039.}	44.6101	0.160198
14	newly 3 weeks spec	8364.	5136.42	{5028., 5252.}	38.589	0.21182	newly 3 weeks cumul	30226.	14436.1	{14280., 14588.}	52.2395	0.320956	newly 5 weeks spec	4850.	5123.25	{4974., 5237.}	5.63402	0.0237238	newly 5 weeks cumul	41750.	24776.8	{24589., 25025.}	40.6544	0.226635	newly 50 weeks cumul	54343.	61708.3	{61298., 62127.}	13.5534	0.0551924
15	newly 3 weeks spec	7352.	4639.05	{4520., 4744.}	36.9008	0.200052	newly 3 weeks cumul	31019.	12504.1	{12361., 12662.}	59.6888	0.394616	newly 5 weeks spec	3380.	5048.07	{4914., 5184.}	49.3512	0.174117	newly 5 weeks cumul	39368.	22503.7	{22243., 22848.}	42.8377	0.242928	newly 51 weeks cumul	45493.	65297.1	{64747., 65776.}	43.5322	0.156941
16	newly 3 weeks spec	8547.	7427.11	{7298., 7544.}	13.1027	0.0610257	newly 3 weeks cumul	32656.	22400.6	{22215., 22544.}	31.4043	0.16371	newly 5 weeks spec	4015.	6028.24	{5926., 6149.}	50.143	0.176459	newly 5 weeks cumul	42788.	35270.8	{35013., 35485.}	17.5685	0.0839126	newly 54 weeks cumul	49793.	60756.8	{60439., 61062.}	22.0188	0.0864231
17	newly 3 weeks spec	6343.	10973.3	{10855., 11103.}	72.9981	0.238023	newly 3 weeks cumul	39322.	33530.6	{33348., 33748.}	14.7283	0.0691992	newly 5 weeks spec	1376.	7893.77	{7772., 8025.}	473.675	0.758638	newly 5 weeks cumul	43673.	50997.	{50763., 51224.}	16.77	0.0673282	newly 60 weeks cumul	44678.	77612.3	{77287., 77825.}	73.7149	0.239835
18	newly 3 weeks spec	6778.	10644.	{10484., 10795.}	57.0378	0.195973	newly 3 weeks cumul	35236.	32208.7	{31985., 32434.}	8.59138	0.0390185	newly 5 weeks spec	2583.	7811.6	{7704., 7922.}	202.424	0.480588	newly 5 weeks cumul	42014.	49393.4	{49156., 49631.}	17.5641	0.0702719	newly 59 weeks cumul	45747.	76288.1	{76133., 76467.}	66.7609	0.222093
19	newly 3 weeks spec	8875.	7166.12	{7065., 7280.}	19.255	0.0929126	newly 3 weeks cumul	35227.	19699.9	{19540., 19843.}	44.0773	0.25242	newly 5 weeks spec	4524.	6915.72	{6789., 7030.}	52.8674	0.184282	newly 5 weeks cumul	46101.	33840.7	{33606., 34089.}	26.5944	0.134277	newly 54 weeks cumul	56361.	71849.2	{71573., 72105.}	27.4804	0.105442
20	newly 3 weeks spec	6233.	10218.8	{10095., 10366.}	63.9461	0.214677	newly 3 weeks cumul	33857.	33065.1	{32786., 33287.}	2.33904	0.010286	newly 5 weeks spec	2013.	6995.33	{6857., 7112.}	247.508	0.540927	newly 5 weeks cumul	39540.	48683.1	{48426., 48945.}	23.1236	0.0903367	newly 59 weeks cumul	42220.	71642.9	{71351., 71911.}	69.6894	0.229653
21	newly 3 weeks spec	6457.	11009.1	{10868., 11137.}	70.4984	0.231698	newly 3 weeks cumul	36224.	33054.1	{32856., 33249.}	8.75083	0.0397764	newly 5 weeks spec	1848.	8064.36	{7946., 8179.}	336.383	0.639836	newly 5 weeks cumul	41535.	50813.2	{50586., 51047.}	22.3382	0.0875592	newly 60 weeks cumul	43675.	78398.3	{78155., 78609.}	79.5037	0.254072
22	newly 3 weeks spec	7395.	8621.13	{8485., 8743.}	16.5805	0.0665875	newly 3 weeks cumul	32127.	29464.4	{29146., 29744.}	8.2877	0.0375861	newly 5 weeks spec	2882.	5663.62	{5568., 5768.}	96.517	0.293355	newly 5 weeks cumul	39687.	42223.9	{41841., 42571.}	6.39222	0.0268991	newly 56 weeks cumul	44378.	60781.6	{60333., 61157.}	36.9633	0.136597
23	newly 3 weeks spec	4881.	8233.02	{8126., 8343.}	68.6749	0.227025	newly 3 weeks cumul	26601.	28915.4	{28765., 29092.}	8.70058	0.0362277	newly 5 weeks spec	1370.	5284.37	{5196., 5363.}	285.72	0.586238	newly 5 weeks cumul	30613.	40888.5	{40725., 41041.}	33.5657	0.125693	newly 57 weeks cumul	31913.	57875.4	{57739., 58035.}	81.3537	0.258526
24	newly 3 weeks spec	7357.	11055.7	{10911., 11210.}	50.275	0.17686	newly 3 weeks cumul	40276.	32145.6	{31934., 32360.}	20.1867	0.0979297	newly 5 weeks spec	2182.	8422.66	{8294., 8542.}	286.006	0.586567	newly 5 weeks cumul	46540.	50530.7	{50252., 50748.}	8.57482	0.0357262	newly 59 weeks cumul	48960.	80085.6	{79883., 80288.}	63.5735	0.213712
25	newly 3 weeks spec	9005.	7932.86	{7812., 8070.}	11.9061	0.0550883	newly 3 weeks cumul	36976.	22788.	{22561., 23006.}	38.3708	0.210227	newly 5 weeks spec	3489.	6763.11	{6652., 6865.}	93.8409	0.287406	newly 5 weeks cumul	46402.	37070.3	{36756., 37390.}	20.1105	0.0975204	newly 55 weeks cumul	51783.	65638.1	{65366., 65893.}	26.756	0.102966
26	newly 3 weeks spec	6466.	7120.7	{7025., 7212.}	10.1253	0.0418595	newly 3 weeks cumul	27532.	22577.	{22426., 22691.}	17.9971	0.0861768	newly 5 weeks spec	2648.	5372.03	{5277., 5482.}	102.871	0.307174	newly 5 weeks cumul	34415.	34242.8	{34030., 34435.}	0.584658	0.00254892	newly 53 weeks cumul	38357.	55339.5	{55038., 55615.}	44.2748	0.159187
27	newly 3 weeks spec	7481.	7524.85	{7407., 7633.}	1.07218	0.00463192	newly 3 weeks cumul	29075.	23623.5	{23481., 23792.}	18.7499	0.0901816	newly 5 weeks spec	4125.	5711.01	{5611., 5792.}	38.4487	0.141256	newly 5 weeks cumul	38835.	36004.3	{35807., 36201.}	7.28902	0.0328728	newly 53 weeks cumul	49190.	58242.8	{57968., 58503.}	18.4037	0.0733626
28	newly 3 weeks spec	5303.	10275.1	{10153., 10393.}	93.7594	0.287243	newly 3 weeks cumul	34205.	32568.3	{32333., 32787.}	4.785	0.0213001	newly 5 weeks spec	965.	7169.63	{7033., 7272.}	642.967	0.870933	newly 5 weeks cumul	37460.	48525.	{48298., 48748.}	29.5383	0.112395	newly 60 weeks cumul	38276.	72527.4	{72349., 72705.}	89.4853	0.277574
29	newly 3 weeks spec	9186.	9623.22	{9504., 9739.}	4.75963	0.0201734	newly 3 weeks cumul	36850.	31082.8	{30929., 31238.}	15.6505	0.0739212	newly 5 weeks spec	4278.	6659.22	{6545., 6761.}	55.662	0.192149	newly 5 weeks cumul	47405.	45907.2	{45712., 46103.}	3.15962	0.0139455	newly 57 weeks cumul	55913.	68357.5	{68206., 68485.}	22.2568	0.0872726
30	newly 3 weeks spec	9408.	11263.4	{11135., 11396.}	19.7213	0.0781544	newly 3 weeks cumul	43928.	32734.6	{32553., 32928.}	25.4814	0.12774	newly 5 weeks spec	3519.	8550.66	{8421., 8676.}	142.986	0.385551	newly 5 weeks cumul	53150.	51416.7	{51136., 51656.}	3.26111	0.0144027	newly 60 weeks cumul	58223.	81121.6	{80852., 81379.}	39.329	0.14404
31	newly 3 weeks spec	6334.	6329.62	{6230., 6424.}	1.03979	0.00452499	newly 3 weeks cumul	24601.	21064.	{20924., 21207.}	14.3773	0.0674171	newly 5 weeks spec	3288.	4611.63	{4515., 4707.}	40.2564	0.146865	newly 5 weeks cumul	32650.	31139.	{30999., 31274.}	4.62799	0.020582	newly 50 weeks cumul	39652.	50317.2	{50068., 50545.}	26.8969	0.103448
32	newly 3 weeks spec	8242.	8306.15	{8198., 8415.}	1.03701	0.00447366	newly 3 weeks cumul	32101.	28855.8	{28704., 28995.}	10.1094	0.046289	newly 5 weeks spec	4308.	5387.99	{5308., 5475.}	25.0694	0.0971197	newly 5 weeks cumul	42469.	41029.2	{40862., 41186.}	3.39017	0.0149806	newly 56 weeks cumul	52787.	58537.6	{58366., 58733.}	10.894	0.0449066
33	newly 3 weeks spec	5633.	9914.68	{9768., 10042.}	76.0107	0.245514	newly 3 weeks cumul	30340.	32970.7	{32712., 33203.}	8.6707	0.036106	newly 5 weeks spec	2012.	6597.24	{6513., 6699.}	227.895	0.515708	newly 5 weeks cumul	35798.	47823.2	{47577., 48039.}	33.5917	0.125776	newly 60 weeks cumul	38479.	69145.7	{68811., 69389.}	79.6973	0.25454
34	newly 3 weeks spec	6383.	7931.59	{7820., 8033.}	24.2612	0.0943098	newly 3 weeks cumul	29792.	27967.1	{27817., 28124.}	6.12547	0.027456	newly 5 weeks spec	2446.	5056.49	{4966., 5138.}	106.725	0.315355	newly 5 weeks cumul	36225.	39439.	{39278., 39571.}	8.8723	0.0369157	newly 56 weeks cumul	39694.	55751.6	{55580., 55904.}	40.4534	0.147531
35	newly 3 weeks spec	5611.	10873.8	{10720., 11036.}	93.7938	0.287314	newly 3 weeks cumul	36155.	32910.4	{32688., 33127.}	8.97406	0.0408396	newly 5 weeks spec	1151.	7907.22	{7784., 8032.}	586.987	0.836919	newly 5 weeks cumul	39920.	50360.	{50090., 50598.}	26.1524	0.100892	newly 60 weeks cumul	40865.	77349.2	{77106., 77550.}	89.2798	0.277103
36	newly 3 weeks spec	9648.	9839.4	{9712., 9962.}	1.99938	0.00857887	newly 3 weeks cumul	37219.	31215.5	{30970., 31431.}	16.1301	0.076401	newly 5 weeks spec	5029.	6957.07	{6839., 7069.}	38.339	0.14091	newly 5 weeks cumul	49093.	46625.6	{46333., 46823.}	5.02591	0.0223984	newly 56 weeks cumul	60543.	70431.9	{70235., 70633.}	16.3337	0.0657047
37	newly 3 weeks spec	8031.	10326.1	{10186., 10452.}	28.5785	0.109146	newly 3 weeks cumul	39903.	28645.8	{28431., 28840.}	28.2115	0.143952	newly 5 weeks spec	2840.	8584.22	{8444., 8703.}	202.261	0.480352	newly 5 weeks cumul	47625.	46990.1	{46724., 47249.}	1.33321	0.00583275	newly 57 weeks cumul	51821.	80000.2	{79816., 80190.}	54.378	0.188585
38	newly 3 weeks spec	6443.	7492.94	{7362., 7624.}	16.2958	0.0655268	newly 3 weeks cumul	29746.	22780.9	{22563., 22973.}	23.4153	0.115872	newly 5 weeks spec	2643.	5951.3	{5825., 6072.}	125.172	0.352463	newly 5 weeks cumul	36633.	35540.5	{35257., 35797.}	2.98234	0.0131587	newly 54 weeks cumul	40381.	59590.3	{59300., 59923.}	47.5702	0.168995
39	newly 3 weeks spec	8056.	8749.67	{8595., 8893.}	8.6106	0.0358406	newly 3 weeks cumul	34505.	28660.1	{28485., 28818.}	16.9393	0.0806086	newly 5 weeks spec	3317.	6089.43	{5978., 6189.}	83.5825	0.263793	newly 5 weeks cumul	43007.	42172.4	{41984., 42312.}	1.94061	0.00851304	newly 56 weeks cumul	48356.	63401.	{63202., 63539.}	31.1129	0.117644
40	newly 3 weeks spec	7422.	8166.93	{8063., 8273.}	10.0368	0.0415143	newly 3 weeks cumul	30770.	28447.1	{28293., 28585.}	7.54917	0.0340931	newly 5 weeks spec	3674.	5278.98	{5173., 5363.}	43.6848	0.157366	newly 5 weeks cumul	39721.	40386.9	{40190., 40588.}	1.67642	0.00721742	newly 57 weeks cumul	48338.	57593.1	{57376., 57767.}	19.1467	0.0760805
41	newly 3 weeks spec	7711.	10976.6	{10850., 11106.}	42.3501	0.153335	newly 3 weeks cumul	45986.	31614.4	{31379., 31832.}	31.252	0.162746	newly 5 weeks spec	1635.	8477.2	{8368., 8576.}	418.483	0.714712	newly 5 weeks cumul	51212.	50067.3	{49819., 50289.}	2.2352	0.00982022	newly 60 weeks cumul	52636.	80141.6	{79919., 80293.}	52.2562	0.182574
42	newly 3 weeks spec	7808.	6871.22	{6755., 6998.}	11.9977	0.0555412	newly 3 weeks cumul	27957.	21959.3	{21767., 22143.}	21.4533	0.104881	newly 5 weeks spec	4650.	5272.78	{5174., 5366.}	13.3931	0.0545411	newly 5 weeks cumul	38862.	33335.2	{33101., 33523.}	14.2216	0.0666273	newly 51 weeks cumul	51353.	56285.9	{56000., 56545.}	9.60583	0.0398306
43	newly 3 weeks spec	8961.	8778.64	{8649., 8905.}	2.0549	0.00904061	newly 3 weeks cumul	34781.	28711.2	{28559., 28866.}	17.4515	0.0832956	newly 5 weeks spec	4598.	6125.09	{6037., 6222.}	33.212	0.124506	newly 5 weeks cumul	46123.	42294.5	{42097., 42477.}	8.30063	0.0376362	newly 56 weeks cumul	56656.	63592.4	{63392., 63846.}	12.243	0.0501578
44	newly 3 weeks spec	7188.	9279.84	{9162., 9402.}	29.1018	0.11091	newly 3 weeks cumul	31836.	30835.3	{30627., 31023.}	3.14327	0.0138749	newly 5 weeks spec	3246.	6242.18	{6126., 6335.}	92.3038	0.283949	newly 5 weeks cumul	39959.	44832.5	{44662., 45050.}	12.1963	0.0499758	newly 56 weeks cumul	46072.	65441.3	{65282., 65600.}	42.0414	0.152414
45	newly 3 weeks spec	7528.	9807.71	{9699., 9930.}	30.2831	0.114865	newly 3 weeks cumul	33385.	32122.2	{31922., 32282.}	3.78248	0.0167503	newly 5 weeks spec	3187.	6644.36	{6546., 6736.}	108.483	0.319042	newly 5 weeks cumul	41374.	46997.6	{46799., 47189.}	13.592	0.0553453	newly 58 weeks cumul	46827.	68895.8	{68700., 69094.}	47.1283	0.167695
46	newly 3 weeks spec	8312.	10482.5	{10362., 10593.}	26.1131	0.100742	newly 3 weeks cumul	39284.	32373.3	{32190., 32524.}	17.5917	0.0840324	newly 5 weeks spec	3124.	7523.22	{7405., 7639.}	140.82	0.38166	newly 5 weeks cumul	47611.	49006.4	{48806., 49221.}	2.93086	0.0125434	newly 60 weeks cumul	52255.	74616.7	{74411., 74774.}	42.7933	0.154707
47	newly 3 weeks spec	8223.	8003.38	{7853., 8149.}	2.68321	0.0118537	newly 3 weeks cumul	34349.	22770.	{22475., 23015.}	33.7098	0.178568	newly 5 weeks spec	3657.	6976.02	{6860., 7093.}	90.758	0.280447	newly 5 weeks cumul	43642.	37412.3	{37074., 37779.}	14.2745	0.0669012	newly 55 weeks cumul	49521.	68080.7	{67770., 68330.}	37.4784	0.138232
48	newly 3 weeks spec	8405.	10278.8	{10121., 10390.}	22.2935	0.0873812	newly 3 weeks cumul	39417.	32794.5	{32568., 32982.}	16.801	0.0798871	newly 5 weeks spec	2763.	7125.69	{7013., 7212.}	157.897	0.41142	newly 5 weeks cumul	47174.	48651.	{48414., 48832.}	3.13086	0.0133862	newly 58 weeks cumul	50734.	72312.6	{72124., 72499.}	42.5327	0.153914
49	newly 3 weeks spec	6054.	7421.95	{7314., 7539.}	22.5958	0.0884465	newly 3 weeks cumul	27886.	26841.5	{26664., 26988.}	3.7455	0.0165859	newly 5 weeks spec	2454.	4633.74	{4547., 4735.}	88.824	0.276006	newly 5 weeks cumul	34341.	37386.1	{37189., 37566.}	8.86724	0.0368912	newly 56 weeks cumul	38035.	51990.3	{51756., 52153.}	36.6907	0.135733
50	newly 3 weeks spec	8264.	8706.88	{8610., 8823.}	5.35915	0.0226465	newly 3 weeks cumul	33965.	30039.7	{29874., 30200.}	11.5569	0.0533408	newly 5 weeks spec	3752.	5668.6	{5562., 5771.}	51.0821	0.179166	newly 5 weeks cumul	43375.	42834.9	{42538., 43066.}	1.24519	0.00544564	newly 57 weeks cumul	50179.	61128.3	{60899., 61471.}	21.8205	0.0857178
